# Biochemical Analysis of the *Plasmodium falciparum* Erythrocyte-binding Antigen-175 (EBA175)-Glycophorin-A Interaction: IMPLICATIONS FOR VACCINE DESIGN*^♦^

**DOI:** 10.1074/jbc.M113.484840

**Published:** 2013-09-16

**Authors:** Madushi Wanaguru, Cécile Crosnier, Steven Johnson, Julian C. Rayner, Gavin J. Wright

**Affiliations:** From the ‡ Cell Surface Signalling Laboratory, Wellcome Trust Sanger Institute, Hinxton, Cambridge CB10 1HH,; the § Malaria Programme, Wellcome Trust Sanger Institute, Hinxton, Cambridge CB10 1SA, and; the ¶ Sir William Dunn School of Pathology, University of Oxford, Oxford OX1 3RE, United Kingdom

**Keywords:** Carbohydrate-binding Protein, Cell Invasion, Cell Surface Receptor, Infectious Diseases, Malaria, Protein-Protein Interactions, Surface Plasmon Resonance (SPR)

## Abstract

*Pf*EBA175 has an important role in the invasion of human erythrocytes by *Plasmodium falciparum* and is therefore considered a high priority blood-stage malaria vaccine candidate. *Pf*EBA175 mediates adhesion to erythrocytes through binding of the Duffy-binding-like (DBL) domains in its extracellular domain to Neu5Acα2–3Gal displayed on the *O*-linked glycans of glycophorin-A (GYPA). Because of the difficulties in expressing active full-length (FL) *P. falciparum* proteins in a recombinant form, previous analyses of the *Pf*EBA175-GYPA interaction have largely focused on the DBL domains alone, and therefore they have not been performed in the context of the native protein sequence. Here, we express the entire ectodomain of *Pf*EBA175 (*Pf*EBA175 FL) in soluble form, allowing us to compare the biochemical and immunological properties with a fragment containing only the tandem DBL domains (“region II,” *Pf*EBA175 RII). Recombinant *Pf*EBA175 FL bound human erythrocytes in a trypsin and neuraminidase-sensitive manner and recognized Neu5Acα2–3Gal-containing glycans, confirming its biochemical activity. A quantitative binding analysis showed that *Pf*EBA175 FL interacted with native GYPA with a *K_D_* ∼0.26 μm and is capable of self-association. By comparison, the RII fragment alone bound GYPA with a lower affinity demonstrating that regions outside of the DBL domains are important for interactions with GYPA; antibodies directed to these other regions also contributed to the inhibition of parasite invasion. These data demonstrate the importance of *Pf*EBA175 regions other than the DBL domains in the interaction with GYPA and merit their inclusion in an EBA175-based vaccine.

## Teaser

**Background:** The GYPA-*Pf*EBA175 interaction is important for erythrocyte invasion by the malaria parasite.

**Results:** The entire ectodomain of EBA175 interacted with GYPA with different biochemical parameters to the previously determined GYPA-binding fragment containing two DBL domains.

**Conclusion:** Regions outside of the tandem DBL domains contribute to GYPA binding by EBA175.

**Significance:** These findings may assist the design of an EBA175-based malaria vaccine.

## Introduction

**FIGURE 1.**
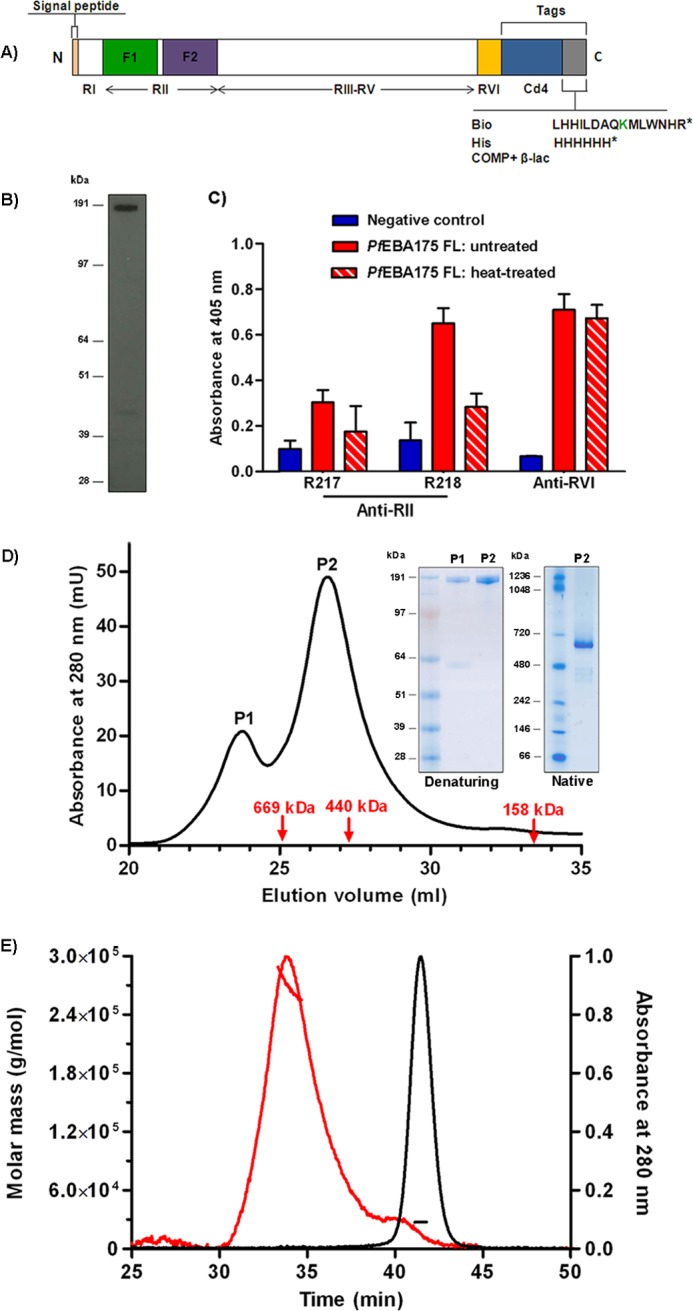
**Recombinant *Pf*EBA175 FL is soluble and immunologically active.** *A,* schematic diagram of *Pf*EBA175 FL highlighting the six regions of homology, RI–RVI, including RII that contains the DBL domains F1 and F2. The sequences are given only for the short peptide tags. *Cd4*, Ig-like domains 3 and 4 of rat Cd4 (∼25 kDa); *Bio,* peptide substrate for the biotin ligase BirA. The biotinylatable lysine residue is indicated in *green. His,* hexa-His. *COMP* + β*-lac,* pentamerization domain of the rat COMP protein and the ampicillin resistance protein β-lactamase. *B,* Western blot of unpurified cell culture supernatant containing biotinylated *Pf*EBA175 FL. *C,* binding by ELISA of three anti-EBA175 monoclonal antibodies to untreated and heat-treated *Pf*EBA175 FL. Negative control is Cd4 tag alone; data are mean ± S.D., *n* = 3. *D,* SEC elution profile of affinity-purified *Pf*EBA175 FL with Coomassie G-250-stained denaturing and native gels of peak fractions (*inset*); *P1,* minor peak; *P2*, major peak. Column void volume = 24 ml; molecular mass standards are indicated with *red arrows. E,* MALS of purified *Pf*EBA175 FL. The peaks correspond to SEC elution (absorbance at 280 nm on the right *y* axis); *horizontal lines* indicate the molecular mass (*left y* axis). *Pf*EBA175 FL is shown in *red* with a 30-kDa monomeric control protein shown for comparison (*black*).

The human malaria parasite, *Plasmodium falciparum,* causes an estimated 300–500 million clinical cases and up to 800,000 deaths each year (1, 2). Widespread implementation of control measures within the last decade, including artemisinin combination therapy and the use of insecticide-treated bed nets, has resulted in a significant decrease in the incidence of *P. falciparum* malaria in endemic countries (3–5). In the face of emerging resistance to artemisinin in parasites and pyrethroid insecticides in mosquito vectors, however, the need for an effective vaccine for long term control and prevention of malaria remains an important global health objective (3, 4, 6). Vaccines that target the blood stage of the infection are conceptually attractive because the parasite is directly exposed to the host humoral immune system, and it is this stage of the life cycle that is responsible for all the clinical symptoms of malaria (7). The blood stage is initiated when an extracellular form of the parasite called the merozoite recognizes and invades host erythrocytes. Invasion is a complex process involving multiple interactions between host erythrocyte receptors and parasite ligands displayed on the merozoite surface. *Pf*EBA175 was the first *P. falciparum* invasion ligand identified and interacts with the highly abundant erythrocyte surface sialoglycoprotein, glycophorin-A (GYPA)^2^ (8–10). *Pf*EBA175 is considered a leading vaccine candidate because antibodies directed against *Pf*EBA175 are present in malaria-immune individuals, and antibodies raised against recombinantly expressed fragments of *Pf*EBA175 inhibit erythrocyte invasion by *P. falciparum in vitro* (11–16). EBA175 is a member of the erythrocyte-binding-like family of *Plasmodium* proteins, which include the Duffy-binding proteins of *Plasmodium vivax* and *Plasmodium knowlesi* (*Pv*DBP and *Pk*DBP) and the *P. falciparum* paralogs EBA140, EBA181, and EBL1. The members of the erythrocyte-binding-like family share a similar gene structure, and this homology has been used to define six regions, RI–RVI, in their ectodomains (Fig. 1*A*) (17). A functional analysis of these regions showed that the RII fragment is capable of rosetting erythrocytes (10). The structure of RII, which comprises two tandem DBL domains (F1 and F2), has been determined by x-ray crystallography (18).

The *Pf*EBA175 receptor, GYPA, is a dimeric type I transmembrane protein carrying 15 closely clustered *O-*linked tetrasaccharides capped with sialic acid/*N*-acetylneuraminic acid (Neu5Ac) (19, 20). The “Neu5Acα2–3Gal” sequence of these glycans was shown to be essential for the recognition of GYPA by *Pf*EBA175 (21). Co-crystallization of the RII fragment with a structural analog of this disaccharide, α-2,3-sialyllactose, revealed six putative glycan-binding sites at the dimer interface of the parasite protein, with four of the sites located within two channels that span the dimer and another two in a deep groove accessible only through a cavity at the top of the dimer (18). Based on the locations of the glycan-binding sites in the RII fragment, and the predominance of the monomeric form of *Pf*EBA175 RII in solution, Tolia and co-workers (22) have proposed a “receptor-induced dimerization model” for the erythrocyte binding of *Pf*EBA175, which postulates monomeric *Pf*EBA175 assembling into a dimer around the dimeric extracellular region of GYPA during invasion. This model is also consistent with recent structural studies investigating the mode of binding of the *Pv*DBP RII to its sulfotyrosine-carrying receptor, DARC.

Although studies of the 68-kDa recombinant *Pf*EBA175 RII have proven highly informative, native *Pf*EBA175 is a much larger protein, being synthesized as a 190-kDa membrane-tethered precursor that is proteolytically cleaved during erythrocyte invasion to release the 175-kDa extracellular region (9). Because of the technical difficulties associated with expressing full-length *Plasmodium* proteins in a functionally active soluble recombinant form (23), much of the biochemical characterization of the *Pf*EBA175-GYPA interaction has been performed using just the RII fragment.

In the study reported here, we expressed the full-length ectodomain of *Pf*EBA175 (*Pf*EBA175 FL) in a soluble recombinant form using a mammalian expression system. We confirmed that the recombinant protein is biochemically active and exhibited binding properties similar to those of native *Pf*EBA175 isolated from parasite cultures. Using this protein, we investigated the biophysical parameters of the EBA175-GYPA interaction and compared them with those of the RII fragment to demonstrate that regions outside the DBL domains contributed to GYPA binding. These data have important implications for the rational design of an effective *Pf*EBA175-based malaria vaccine.

## EXPERIMENTAL PROCEDURES

### 

#### 

##### Recombinant Expression and Purification of Proteins

The sequence encoding the entire extracellular domain of *P. falciparum* (3D7) EBA175 (*Pf*EBA175 FL) except the signal peptide (amino acids 21–1424) was made by gene synthesis (GeneART). The codons were optimized for expression in human cells, and the potential *N*-linked glycosylation sites were removed as described (24). The sequence coding for RII of *Pf*EBA175 (amino acids 142–764) was amplified from this construct by PCR. The coding sequences were cloned into pTT3-derived vectors using unique flanking NotI and AscI restriction enzyme sites, between the leader sequence of the mouse variable κ light chain 7–33 (25) and a rat Cd4 (Ig-like domains 3 and 4) tag, followed either by an enzymatically biotinylatable peptide tag, the pentamerization domain of the rat cartilage oligomeric matrix protein (COMP), and ampicillin resistance protein β-lactamase, or a hexa-His tag (24, 26). The proteins were expressed by transient transfection of HEK293 cells grown in suspension culture as described (27, 28) and collected from the cell culture supernatant 6 days post-transfection. Biotinylation of proteins was achieved by co-transfection with a secreted form of the *Escherichia coli* biotin ligase, BirA (27), and excess unconjugated d-biotin was removed by extensive dialysis into HBS. His-tagged proteins were purified from the culture supernatants by affinity chromatography on HisTrap HP columns (GE Healthcare) using an ÄKTAxpress (GE Healthcare) according to the manufacturer's instructions. Size exclusion chromatography (SEC) of nickel-purified proteins was carried out on a Superdex 200 Tricorn 10/600 column (GE Healthcare) in HBS-EP (HBS, 3 mm EDTA, 0.005% v/v Surfactant P20 (GE Healthcare).

##### Western Blotting

Proteins were resolved under reducing conditions, blotted, and detected using horseradish peroxidase (HRP)-conjugated extravidin (Sigma) essentially as described (24).

##### Enzyme-linked Immunosorbent Assays (ELISA)

Biotinylated proteins were detected by ELISA essentially as described (24). Commercially available glycophorin-A preparations (catalog numbers: G7903 and A9791, Sigma) were biotinylated *in vitro* by incubation with a 20-fold molar excess of EZ-link sulfo-NHS-biotin (Thermo Scientific) for 30 min at room temperature and dialyzed into HBS prior to its use in the assays. When testing for immunoreactivity, proteins were immobilized with or without prior treatment at 80 °C for 10 min.

##### Erythrocyte Binding Assays

Biotinylated *Pf*EBA175 and Cd4 (negative control) were multimerized by immobilization on streptavidin-coated Nile Red fluorescent 0.4–0.6-μm microbeads (Spherotech Inc.) by incubation for 45 min at 4 °C (29). The bead arrays were then presented to erythrocytes in flat-bottomed 96-well microtiter plates at a density of ∼3 × 10^5^ cells/well (the ratio of cells/beads = 1:200). After incubation for 1 h at 4 °C with the protein arrays, the cells were washed twice in HBS + 1% BSA (HBS/BSA) and analyzed by flow cytometry. The data were acquired on an LSRII cytometer (BD Biosciences) using the FACS Diva software (BD Biosciences). Nile Red was excited by a blue laser and detected with a 575/26 filter. Forward scatter and side scatter voltages of 430 and 300 V, respectively, and a threshold of 26,100 on forward scatter were applied when analyzing erythrocytes. To test for binding specificity, the erythrocytes were pretreated with either tosylsulfonylphenylalanyl chloromethyl ketone-treated trypsin from bovine pancreas (Sigma) (at 0.25, 0.5, and 1 mg/ml), tosyl-lysyl chloromethyl ketone-treated chymotrypsin from bovine pancreas (Sigma) (at 0.25, 0.5 and 1 mg/ml), or 0.1 milliunit/10^6^ cells of *Vibrio cholerae* neuraminidase (Sigma), for 1 h at 37 °C. Trypsin- and chymotrypsin-treated cells were washed once, treated with 0.5 mg/ml soybean trypsin-chymotrypsin inhibitor (Sigma) for 10 min at room temperature, and then washed twice more before incubation with *Pf*EBA175-coated beads. Neuraminidase-treated cells were washed twice prior to use in the binding assays.

##### Modified Avidity-based Extracellular Interaction Screening (AVEXIS) Assays

The ELISA-based AVEXIS methodology, as described previously (27), was adapted for detecting the interactions of recombinant *Pf*EBA175 proteins, expressed as β-lactamase-tagged pentameric “preys” with biotinylated ‘bait’ forms of purified GYPA and synthetic carbohydrate probes (GlycoTech). Briefly, the baits and the preys were normalized to activities that have previously been shown to detect transient interactions (monomeric half-lives less than 0.1 s). The biotinylated baits were immobilized on streptavidin-coated 96-well microtiter plates (NUNC) at concentrations sufficient for the complete saturation of the available binding surface/well. The plates were then washed twice in HBST and blocked with HBS/BSA for 0.5–1 h, before addition of normalized β-lactamase-tagged prey proteins and incubation for 2 h. After washes in HBST and HBS, 125 μg/ml nitrocefin, a β-lactamase substrate, was added to the wells, and its hydrolysis was monitored by absorbance measurements at 485 nm on a Pherastar plus (BMG Laboratories). All steps were performed at room temperature.

##### Lectin Binding Assay with Purified GYPA

Biotinylated lectins (Vector Laboratories) were immobilized on streptavidin-coated 96-well microtiter plates (NUNC) at 10 μg/ml for 1 h. The plates were then washed twice in HBST and blocked with HBS/BSA for 30 min. The immobilized lectins were next incubated with 0.02 mg/ml purified GYPA (Sigma) for 2 h. After washes in HBST, the plates were incubated with 1 μg/ml of the anti-GYPA mouse monoclonal antibody BRIC256 (Abcam) for 1 h, followed by an alkaline phosphatase-conjugated anti-mouse secondary antibody (Sigma) for 30 min, before the addition of *p*-nitrophenyl phosphate (Sigma) at 1 mg/ml and measuring the absorbance at 405 nm on a Pherastar Plus (BMG Laboratories). All steps were performed at room temperature.

##### Surface Plasmon Resonance (SPR)

SPR studies were performed on a BIAcore T100 instrument (GE Healthcare) at 37 °C, using HBS-EP (GE Healthcare) as the running buffer. In each experiment, biotinylated baits were immobilized on streptavidin-coated sensor chips (GE Healthcare), with the negative control bait in the “reference” flow cell and an approximate molar equivalent amount of the “query” baits in the other flow cells. Purified analyte proteins were separated by size exclusion chromatography on a Superdex 200 Tricorn 10/600 column just before their use in the SPR experiments. Increasing concentrations of these proteins were injected over the immobilized baits at 20 μl/min for equilibrium measurements and at 100 μl/min for kinetic measurements. The surfaces were regenerated with a pulse of 5 m NaCl at the end of each injection cycle. Duplicate injections of the same concentration in each experiment were superimposable, demonstrating no loss of activity after surface regeneration. Reference-subtracted sensorgrams were analyzed using the BIAcore evaluation software version 1.1.1 (GE Healthcare). To determine the overall equilibrium binding affinity, binding responses in the steady-state region of the sensorgrams (*R*_eq_) were plotted against analyte concentration (*C*) and fitted to the following equation: *R*_eq_ = *CR*_max_/(*C* + *K_D_*), where *R*_max_ is the maximum binding response, and *K_D_* is the equilibrium dissociation constant. Kinetic constants were calculated by nonlinear regression fitting to the association and dissociation phases of the sensorgrams. To identify the mechanism of binding, the sensorgrams were globally fitted to three predefined interaction models as follows: simple 1:1 binding (*A* + *B* ↔ *AB*, where *A* is the soluble analyte and *B* is the immobilized ligand); conformational change (*A* + *B* ↔ *AB* ↔ AB*); and bivalent analyte (*AA* + *B* ↔ *AAB*; *AAB* + *B* ↔ *AABB*).

##### Multiangle Light Scattering Measurements (MALS)

Size exclusion chromatography was performed on Superose 6 10/30 (*Pf*EBA175 FL) and Superdex 200 10/30 (*Pf*EBA175 RII) columns (GE Healthcare) equilibrated in HBS (GE Healthcare) at 0.4 ml/min. The column was followed in line by a Dawn Heleos-II light scattering detector (Wyatt Technologies) and an Optilab-Rex refractive index monitor (Wyatt Technologies). Molecular mass calculations were performed using ASTRA 5.3.4.14 (Wyatt Technologies) assuming a d*n*/d*c* value of 0.186 ml/g.

##### P. falciparum Culture and Invasion Assays

The 3D7 and Dd2 strains of *P. falciparum* were cultured in human O+ erythrocytes at 5% hematocrit in complete medium (RPMI 1640 medium containing 10% human serum), under an atmosphere of 1% O_2_, 3% CO_2_, and 96% N_2_. Invasion assays were performed as described previously (30).

##### Polyclonal Antibodies

To raise polyclonal sera against *Pf*EBA175 FL and RII, the purified proteins were injected into rabbits (Cambridge Research Biochemicals, Billingham, UK). The sera were purified on HiTrap Protein G HP columns (GE Healthcare) using an ÄKTAxpress (GE Healthcare) and dialyzed into RPMI 1640 medium (Invitrogen) prior to their use. The anti-*Pf*EBA175 FL and anti-*Pf*EBA175 RII antibodies had similar anti-Cd4 activity, as determined by ELISA. An anti-AMA1 polyclonal (31) was used in the invasion assays as a control.

## RESULTS

### 

#### 

##### Expression of a Soluble, Biochemically Active Full-length Ectodomain of PfEBA175 (PfEBA175 FL)

The technical challenges associated with expressing full-length *Plasmodium* proteins in a recombinant, biochemically active form (23) have prevented a detailed biochemical investigation of the EBA175-GYPA interaction, with most studies limited to a fragment of EBA175 encompassing the two tandem DBL domains (RII). To investigate the biochemical properties and functional activity of a more physiologically relevant *Pf*EBA175 protein, we took advantage of a strategy utilizing a mammalian expression system and codon-optimized gene sequences that had previously been successful for producing biochemically active *Plasmodium* proteins (24, 26). Using this method, we expressed the entire full-length ectodomain fragment of *Pf*EBA175 (*Pf*EBA175 FL) from the 3D7 isolate of *P. falciparum* as a C-terminally tagged soluble fusion protein (Fig. 1*A*). Western blotting of unpurified culture supernatants confirmed the presence of a protein at the expected size (Fig. 1*B*). To determine whether the recombinant *Pf*EBA175 FL was correctly folded and biochemically active, its immunoreactivity to two mouse monoclonal antibodies, R217 and R218, raised against the RII fragment of EBA175 expressed in Sf21 insect cells and known to bind to nonlinear heat-labile epitopes within *Pf*EBA175 RII, were tested (32). Immunoreactivity to *Pf*EBA175 FL was observed for both R217 and R218. Importantly, this immunoreactivity was heat-labile suggesting that at least the epitopes recognized by R217 and R218 are correctly folded in the recombinant protein (Fig. 1*C*). We were also able to demonstrate immunoreactivity to other parts of the EBA175 protein using a mouse monoclonal antibody raised against a yeast-expressed *Pf*EBA175 RVI fragment (33). This antibody, however, showed similar binding to both untreated and heat-treated samples of *Pf*EBA175 FL, suggesting that it recognizes a nonheat-labile linear epitope (Fig. 1*C*).

##### PfEBA175 FL Is Primarily Monomeric with a Small Amount of Self-association

The crystal structure of RII has shown that EBA175 may function by dimerization (18), and so to assess the oligomeric state of *Pf*EBA175 FL, the purified protein was analyzed by SEC. The elution profile of *Pf*EBA175 consisted of two peaks, both containing the protein of interest as demonstrated by denaturing SDS-PAGE. The smaller peak eluted in the void volume of the column, and the size of the major peak was estimated to be ∼500 kDa. A similar mass for *Pf*EBA175 FL was observed by native PAGE, consistent with it being primarily homodimeric in solution (Fig. 1*D*). To further investigate the oligomeric state of *Pf*EBA175 FL, it was subjected to MALS immediately following SEC. This analysis revealed that the main peak of *Pf*EBA175 FL is predominantly monomeric with a certain degree of self-association (Fig. 1*E*). The early elution of *Pf*EBA175 FL in SEC and its retarded mobility in native PAGE may therefore be due to a large hydrodynamic shape, possibly due to a high degree of conformational flexibility or an elongated structure.

##### PfEBA175 FL Binds Human Erythrocytes in a Neuraminidase- and Trypsin-sensitive but Chymotrypsin-resistant Manner

**FIGURE 2.**
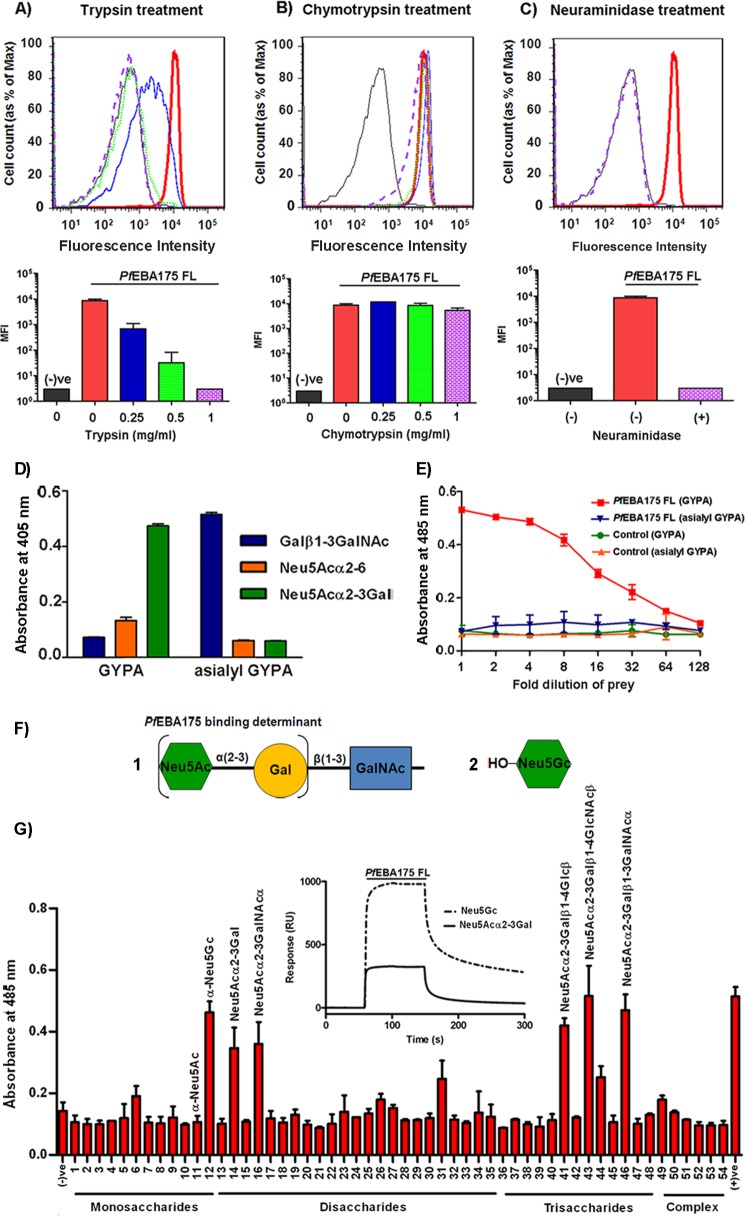
**Recombinant *Pf*EBA175 FL exhibits similar biochemical properties to native *Pf*EBA175 purified from parasite cultures.** *A–C, Pf*EBA175 FL-coated fluorescent beads were presented to untreated human erythrocytes or erythrocytes pre-treated with different concentrations of trypsin (*A*), chymotrypsin (*B*), or neuraminidase (*C*), and bead binding was quantified by cell-associated fluorescence intensity. Negative control was Cd4-coated beads. The *bar charts* represent the median fluorescence intensity (*MFI*) of the cell populations at the same wavelength, as mean ± S.D., *n* = 3. *D,* ELISA demonstrating the glycan composition of native and an asialylated derivative of GYPA using three lectins as follows: *Arachis hypogaea* (binds nonsialylated Galβ1–3GalNAc), *Sambucus nigra* (terminal Neu5Acα2–6), and *Maackia amurensis* (Neu5Acα2–3Gal). *E,* recombinant pentamerized β-lactamase-tagged *Pf*EBA175 FL bound GYPA but not asialyl-GYPA. Negative control was pentameric β-lactamase-tagged Cd4. *D* and *E,* data points are mean ± S.D., *n* = 3. *F,* schematic representation of the following: *1,* a monosialylated (*i.e.* Neu5Ac carrying) human *O-*linked glycan; *2*, Neu5Gc. Neu5Gc has an additional hydroxyl group in comparison with Neu5Ac. *G,* pentameric β-lactamase-tagged *Pf*EBA175 FL was screened for interactions against a panel of 54 carbohydrate probes (Table 1). Positive control = monoclonal antibody (OX68) capturing *Pf*EBA175 FL by its Cd4 tag; negative control = linker region common to all glycans; data points are mean ± S.D., *n* = 2. *Inset,* reference-subtracted SPR sensorgrams showing the binding of *Pf*EBA175 FL as an analyte to immobilized Neu5Gc and Neu5Acα2–3Gal; β-d-glucose was used as the reference.

To determine whether recombinant *Pf*EBA175 FL is biochemically active, we first asked whether it could bind human erythrocytes in a manner consistent with its recognition of GYPA. Interactions between cell surface proteins typically have low binding affinities, and multimerized, highly avid binding reagents are often required to facilitate their detection (34). Therefore, to estimate the degree to which the *Pf*EBA175 FL protein could associate with human erythrocyte surfaces, we first created a highly avid *Pf*EBA175 FL binding reagent by clustering the biotin-tagged monomeric EBA175 protein around fluorescent streptavidin-coated beads. The EBA175-coated beads were then presented to human erythrocytes, and the extent to which they bound was quantified by fluorescence-activated cell sorting. We observed that *Pf*EBA175 FL-coated beads were clearly able to bind to human erythrocytes relative to the negative control (Fig. 2, *A–C*). The binding of EBA175 to erythrocytes is known to be sensitive to the treatment of the cells with the enzymes trypsin and neuraminidase but is insensitive to chymotrypsin (8). Therefore, to assess the specificity of the binding of *Pf*EBA175 FL-coated beads, erythrocytes were pretreated with these enzymes. Treatment with trypsin reduced the binding of *Pf*EBA175 FL to erythrocytes in a dose-dependent manner (Fig. 2*A*), whereas a similar treatment with chymotrypsin had no significant effect, even at the highest concentration (Fig. 2*B*). Pretreatment of the erythrocytes with *V. cholerae* neuraminidase, which preferentially cleaves α2–3-linked sialic acids of *O-*linked tetrasaccharides, was sufficient to prevent all EBA175 binding (Fig. 2*C*).

##### PfEBA175 FL Binds Human Native GYPA and Neu5Acα2–3Gal-containing Glycans

To determine whether recombinant *Pf*EBA175 FL bound native GYPA directly, we used a preparation of GYPA extracted from human erythrocytes. First, we characterized the glycan profile of the native GYPA preparation using three lectins with known carbohydrate-recognition specificities, which revealed a predominance of Neu5Acα2–3Gal with a much smaller amount of Neu5Acα2–6-linked glycans (Fig. 2*D*). An asialylated form of GYPA included as a control appeared to mainly carry Galβ1–3GalNAc (T antigen), as expected. To detect the direct binding of *Pf*EBA175 FL to native GYPA, we chemically biotinylated GYPA and captured it on streptavidin-coated plates before incubating this with *Pf*EBA175 FL expressed as a highly avid β-lactamase-tagged pentamer (27). Using this approach, pentamerized *Pf*EBA175 FL showed saturable binding to GYPA, but not to its asialylated derivative demonstrating that the recombinant *Pf*EBA175 antigen associates directly with GYPA in a “Neu5Acα2–3Gal”-dependent manner (Fig. 2*E*).

The expression of the entire ectodomains of EBA175 as a highly avid recombinant protein enabled us to systematically assess the glycan binding properties of EBA175 by screening a large panel of 54 synthetic carbohydrates, primarily selected because they or their close derivatives are known to be present at the surface of human erythrocytes (Fig. 2, *F* and *G,* and Table 1). All oligosaccharides containing the Neu5Acα2–3Gal determinant were recognized by *Pf*EBA175 FL (Fig. 2*G*). Previous work has suggested that the glycan binding properties of *Pf*EBA175 may be responsible for the restriction of *P. falciparum* to humans because it has been reported that *Pf*EBA175 is unable to bind Neu5Gc (*N*-glycolylneuraminic acid) (35), the hydroxylated form of Neu5Ac that is absent from human erythrocytes but present in other great apes (36). Interestingly, we observed clear binding of *Pf*EBA175 FL to the Neu5Gc monosaccharide in the screen, and this was subsequently confirmed by SPR (Fig. 2*G*). Together, these data demonstrate that the recombinant soluble protein consisting of the entire ectodomain of *Pf*EBA175 is biochemically active and can directly bind native GYPA in a Neu5Acα2–3Gal-dependent manner.

**TABLE 1:**
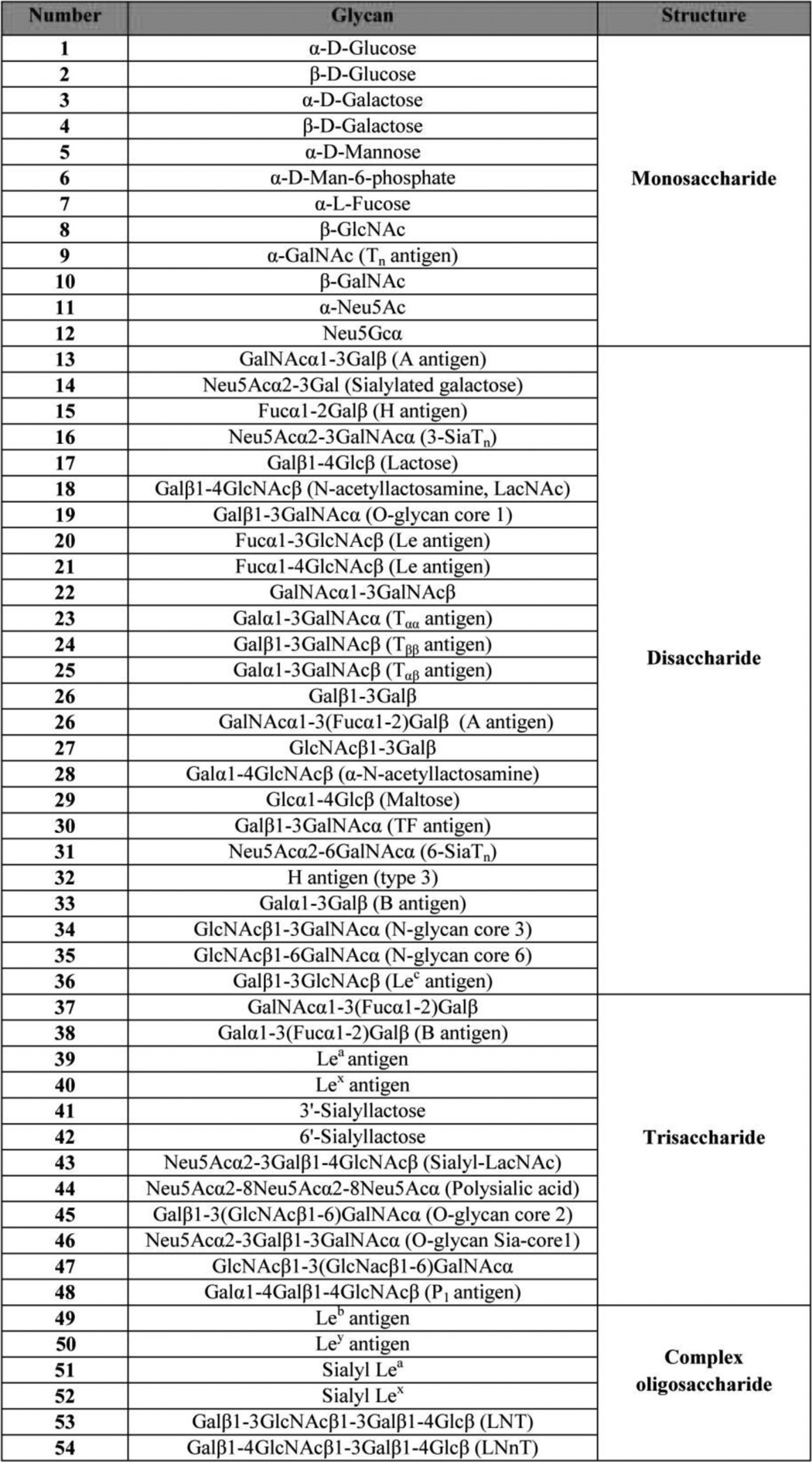
**List of the detailed contents of the human erythrocyte synthetic carbohydrate panel**

##### PfEBA175 FL and GYPA Directly Interact with a K*_D_* of ∼0.26 μm

**FIGURE 3.**
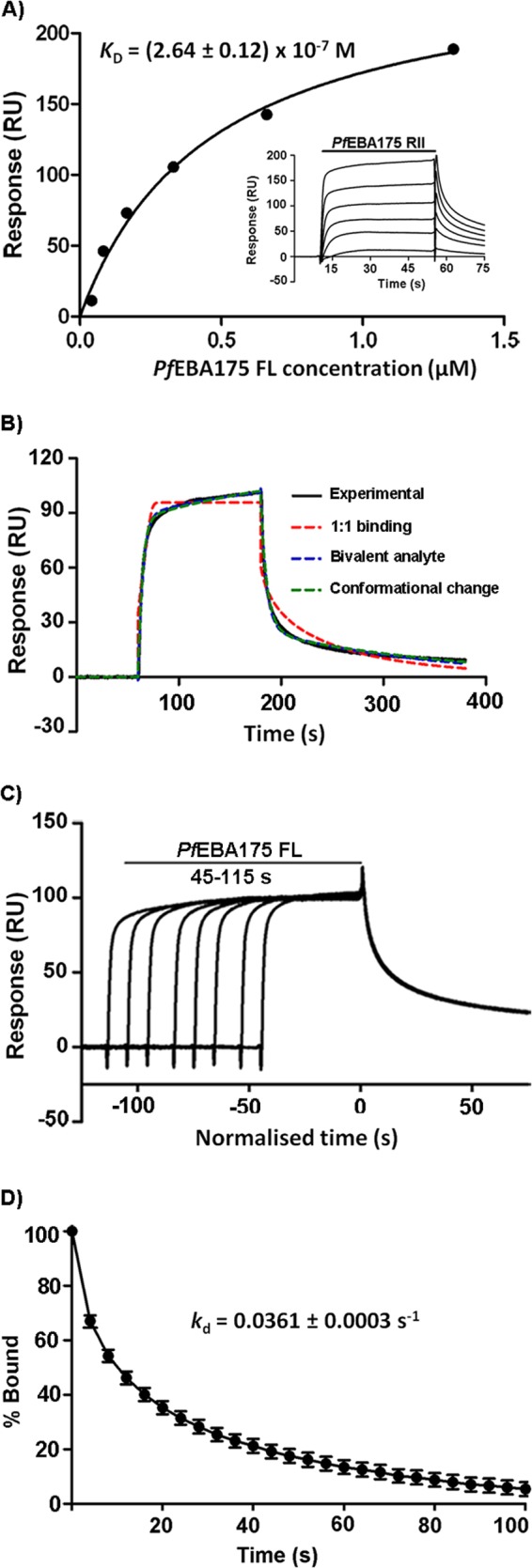
***Pf*EBA175 FL binds sialylated GYPA with a *K_D_* of 0.26 μm.** *A,* SPR analysis of the binding of *Pf*EBA175 FL to GYPA. 2-Fold dilutions of purified *Pf*EBA175 FL were injected over biotinylated GYPA immobilized on a streptavidin-coated sensor chip until equilibrium had been reached (shown in the *inset*). Reference-subtracted binding data were plotted as a binding curve, and the *K_D_* was calculated by global fitting to a steady-state 1:1 interaction model. *K_D_* is shown as mean ± S.E., based on two independent experiments. Biotinylated Cd4 tag was used as the reference. *B,* fits of a *Pf*EBA175 FL-GYPA sensorgram to three predefined kinetic models. An experimental curve is shown overlaid with the fitted curves from the 1:1 binding (χ^2^ = 7.98), bivalent analyte (χ^2^ = 0.642), and conformational change (χ^2^ = 0.763) models. *C,* reference-subtracted sensorgrams obtained by injecting 0.33 μm of *Pf*EBA175 FL over GYPA for various lengths of time (45–115 s) showed no alteration in the dissociation rate suggesting no detectable conformational changes of EBA175 upon binding. *D,* serial dilutions of soluble *Pf*EBA175 FL were injected over biotinylated *Pf*EBA175 FL immobilized on an SPR sensor chip and shown to interact. The dissociation phase data from the reference-subtracted sensorgrams is shown as mean ± S.D., *n* = 3; for clarity, only every 40th data point is shown.

The ability to express and purify *Pf*EBA175 FL enabled us to investigate its interaction with native GYPA in detail. We first determined the biophysical properties of the *Pf*EBA175 FL-GYPA interaction using SPR as implemented in a BIAcore instrument. Affinity purified *Pf*EBA175 FL was first resolved by SEC, and fractions encompassing the main peak were pooled and serial dilutions injected over GYPA immobilized on a sensor chip. Binding of *Pf*EBA175 FL to native GYPA was observed and quantified once equilibrium had been reached to derive an equilibrium dissociation constant (*K_D_*) of ∼0.26 μm (Fig. 3*A*). Although this interaction is relatively weak as expected, it is still ∼4-fold stronger than the two other *P. falciparum* ligand-erythrocyte receptor interactions characterized in our laboratory, *Pf*RH5-Basigin and *Pf*MTRAP-Semaphorin 7A, each of which has a *K_D_* of ∼1 μm (24, 26).

##### Kinetic Analysis of the PfEBA175 FL-GYPA Interaction

Previous research using fragments of the *Pf*EBA175 protein has hypothesized that EBA175 interacts with GYPA in a two-stage process, involving receptor-induced dimerization (18) and a conformational change in the EBA175 protein (37, 38). To characterize the mechanistic details of the interaction between GYPA and the entire *Pf*EBA175 ectodomain, we performed a kinetic analysis using *Pf*EBA175 FL. The association and dissociation kinetic rate constants, *k_a_* and *k_d_*, were determined using nonlinear curve fitting to a set of reference-subtracted sensorgrams using the initial binding and wash-out phases. Both the *k_a_* (8.6 ± 0.2 × 10^5^ m^−1^ s^−1^) and *k_d_* (0.09682 ± 0.00008 s^−1^) values were typical for a relatively weak protein-protein interaction. To gain a better mechanistic understanding of the interaction, the binding data were fitted to three models (Fig. 3*B*). The fits to the conformational change and bivalent analyte models were similar and better than that of the simple 1:1 binding model (Fig. 3*B*). To test whether a conformational change was involved in the binding of *Pf*EBA175 FL to GYPA, we injected *Pf*EBA175 FL until saturation was achieved for a range of different contact times. We observed that variations in contact time did not influence the dissociation phase, suggesting that the interaction of *Pf*EBA175 FL with GYPA does not involve a slow, temporally resolvable conformational change that stabilizes the complex (Fig. 3*C*). Using SEC-MALS, we established that *Pf*EBA175 FL was primarily monomeric in solution albeit with some self-association. To test the relevance of the bivalent analyte model for the *Pf*EBA175-GYPA interaction, we analyzed the propensity of *Pf*EBA175 FL to interact with itself by SPR. We quantified the observed homophilic binding using the dissociation phase of the interaction to avoid the confounding problem of analyte self-association, which would lead to an underestimate of the affinity in equilibrium analyses (Fig. 3*D*). *Pf*EBA175 FL self-associated with a *k_d_* value (∼0.04 s^−1^), consistent with a role for EBA175 dimerization in its interaction with GYPA.

##### PfEBA175 RII Interacts with GYPA with a 10-Fold Lower Affinity than PfEBA175 FL

**FIGURE 4.**
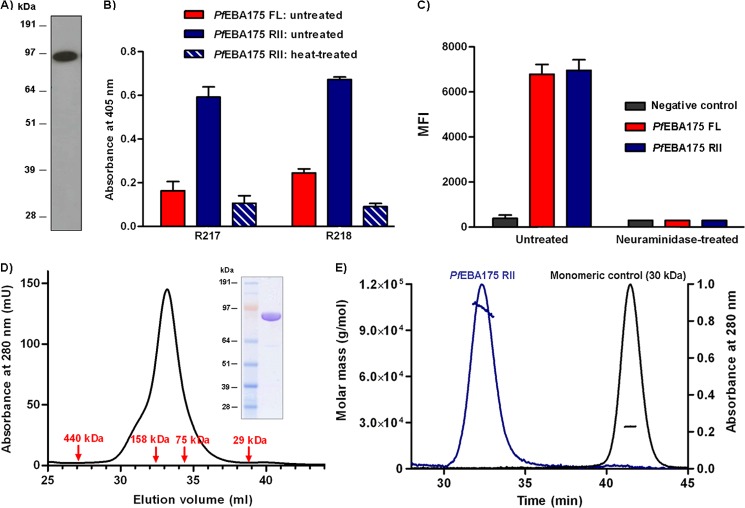
**Biochemically active recombinant *Pf*EBA175 RII is largely monomeric in solution.** *A,* Western blot of unpurified cell culture supernatant containing biotinylated *Pf*EBA175 RII. *B,* ELISA demonstrating immunoreactivity of two anti-EBA175 RII mouse monoclonal antibodies to untreated and heat-treated *Pf*EBA175 RII and untreated *Pf*EBA175 FL. *C,* median fluorescence intensities (*MFI*) associated with untreated and neuraminidase-treated erythrocytes incubated with fluorescent beads coated with Cd4, *Pf*EBA175 FL, or *Pf*EBA175 RII. *B* and *C*, *bar charts* show mean ± S.D., *n* = 3. *D,* SEC profile of affinity-purified His-tagged *Pf*EBA175 RII and a Coomassie G-250-stained denaturing gel of the peak fraction (*inset*). *E,* multiangle light scattering of purified *Pf*EBA175 RII (*blue line*) and a control monomeric protein of 30 kDa used as a reference (*black line*). The peaks correspond to SEC elution with the absorbance at 280 nm on the *right y* axis. The *horizontal lines* indicate the molecular mass (*left y* axis).

The fragment of EBA175 containing the tandem DBL domains (RII) is known to bind GYPA (10); however, whether other regions of EBA175 also contribute to erythrocyte recognition is not well established (12, 37, 38). To compare the binding properties of the RII fragment with the full-length *Pf*EBA175, we also expressed RII as a soluble, recombinant form using HEK293 cells. A biochemical characterization of the *Pf*EBA175 RII protein revealed that it was expressed at the expected size, was recognized by the conformation-sensitive monoclonal antibodies R217 and R218, and bound human erythrocytes in a neuraminidase-sensitive manner suggesting that it is correctly folded (Fig. 4, *A–C*). *Pf*EBA175 RII eluted as a monodisperse peak at ∼125 kDa when analyzed by SEC consistent with it adopting a primarily monomeric form in solution (Fig. 4*D*). Further analysis by MALS confirmed that *Pf*EBA175 RII, similar to *Pf*EBA175 FL, was primarily monomeric, but it showed some propensity to self-associate (Fig. 4*E*).

**FIGURE 5.**
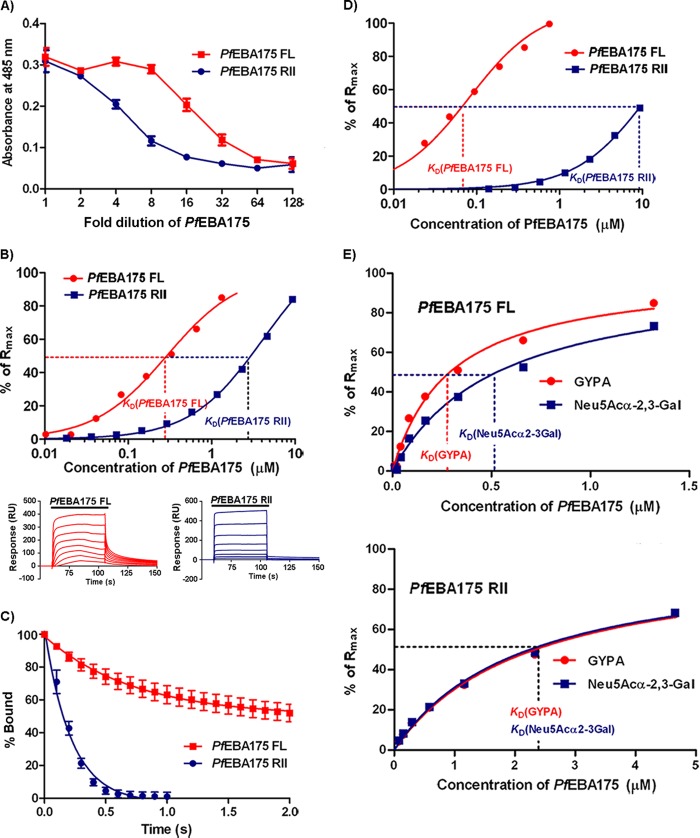
***Pf*EBA175 RII binds GYPA with an ∼10-fold lower affinity than *Pf*EBA175 FL.** *A,* pentameric β-lactamase-tagged *Pf*EBA175 FL and *Pf*EBA175 RII were normalized, serially diluted, and tested for binding to GYPA. *B,* 2-fold serial dilutions of *Pf*EBA175 FL and *Pf*EBA175 RII were injected over immobilized GYPA and Cd4 (reference) until equilibrium was reached (as shown in *insets below*). Reference-subtracted binding data were plotted as a binding curve. *Pf*EBA175 RII bound GYPA with a *K_D_* of (25.25 ± 0.85) × 10^−7^ m (mean ± S.E., from two independent experiments), 10-fold weaker than that of *Pf*EBA175 FL. *C, Pf*EBA175 RII dissociates faster than *Pf*EBA175 FL following binding to GYPA. The dissociation phase data from the reference-subtracted sensorgrams are shown as mean ± S.D., *n* = 3. *D, Pf*EBA175 RII bound *Pf*EBA175 FL with much weaker affinity than *Pf*EBA175 FL itself. Serial dilutions of *Pf*EBA175 FL and *Pf*EBA175 RII were injected over immobilized *Pf*EBA175 FL, and reference-subtracted binding data were used to estimate the *K_D_* values. The responses at equilibrium are shown as a fraction of the calculated *R*_max_. *E, Pf*EBA175 FL and *Pf*EBA175 RII bind Neu5Acα2–3Gal with *K_D_* values of (5.15 ± 0.75) × 10^−7^ m and (24.0 ± 2.7) × 10^−7^ m respectively. 2-Fold dilutions of *Pf*EBA175 FL and *Pf*EBA175 RII were injected across immobilized GYPA and Neu5Acα2–3Gal using Cd4 and β-d-glucose as the references, respectively.

We used a pentamerized enzyme-tagged *Pf*EBA175 RII protein to show that it could bind GYPA, although in comparison with the normalized *Pf*EBA175 FL control, the interaction detected using this fragment was more sensitive to dilution of the protein, suggesting it had a lower affinity for GYPA (Fig. 5*A*). Using SPR, we quantified this binding and found that *Pf*EBA175 RII bound GYPA with a *K_D_* of ∼2 μm, ∼10-fold weaker in comparison with the *Pf*EBA175 FL-GYPA interaction (Fig. 5*B*). Consistent with this, a comparative kinetic analysis showed that the weaker interaction strength of *Pf*EBA175 RII was due to a faster dissociation rate, ruling out the possibility that a significant fraction of the *Pf*EBA175 RII protein was functionally inactive (Fig. 5*C*). One reason for the weaker binding of the RII fragment to GYPA could be due to a reduced ability to self-associate. To examine this, we compared the binding affinity of the RII fragment and the full-length ectodomain to *Pf*EBA175 FL and found that the former interacted with an ∼70-fold weaker binding affinity (Fig. 5*D*). It has been previously shown that both the GYPA peptide backbone and sialic acid are required for EBA175 binding (10). To determine whether these binding properties could be distinguished and/or attributed to different regions of EBA175 protein, we directly compared the binding of both *Pf*EBA175 FL and *Pf*EBA175 RII analytes to GYPA and the oligosaccharide Neu5Acα2–3Gal. Binding of *Pf*EBA175 RII to GYPA and Neu5Acα2–3Gal was indistinguishable from one another, whereas *Pf*EBA175 FL bound GYPA with ∼2-fold higher affinity than for the glycan alone (Fig. 5*E*). These results suggest that *Pf*EBA175 RII interacts with the glycan moieties of GYPA but that the extracellular regions of EBA175 outside of RII also contact the polypeptide backbone of GYPA, contributing further binding energy to the interaction.

##### Comparison of PfEBA175 FL and PfEBA175 RII as Vaccine Candidates

**FIGURE 6.**
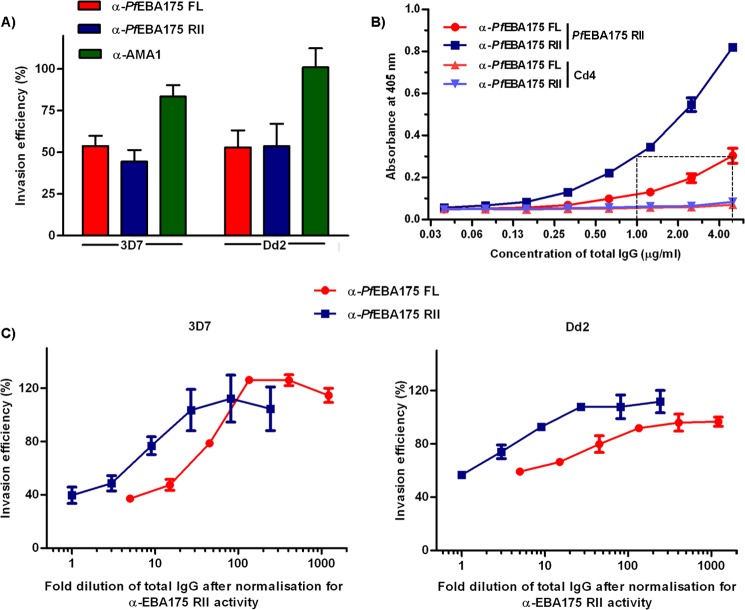
**Antibodies directed against the extracellular regions of *Pf*EBA175 outside of RII can inhibit erythrocyte invasion by both sialic acid-dependent and -independent strains of *P. falciparum*.** *A,* rabbit polyclonal antibodies raised against *Pf*EBA175 FL and *Pf*EBA175 RII inhibit erythrocyte invasion by the *P. falciparum* strains 3D7 (sialic acid-independent) and Dd2 (sialic acid-dependent). An anti-AMA1 3D7 polyclonal antibody was included in the experiment as a control; all antibodies were purified and used at 2.5 mg/ml. Invasion efficiency was estimated relative to a no antibody control. *B,* binding of anti-*Pf*EBA175 FL and anti-*Pf*EBA175 RII polyclonal antibodies to biotinylated *Pf*EBA175 RII as determined by ELISA. Prior to performing the assay, antibodies against the Cd4 tag were removed from the polyclonal sera by pre-adsorption. The anti-*Pf*EBA175 FL sera contained ∼5-fold lower immunoreactivity to RII, relative to the anti-*Pf*EBA175 RII sera. *C,* inhibition of erythrocyte invasion by anti-*Pf*EBA175 FL and anti-*Pf*EBA175 RII sera normalized for anti-RII activity. All data are shown as mean ± S.D., *n* = 3.

Given the important role of EBA175 in erythrocyte invasion and the early success in expressing region II as an active recombinant protein, this fragment has been advanced as a potential malaria vaccine (12, 39). Because we found that regions of EBA175 outside of the tandem DBL domains influenced its ability to interact with GYPA, we asked whether PfEBA175 FL would be able to elicit a more potent invasion-blocking antibody response than the RII fragment alone. To address this, rabbits were immunized with an equal mass of both proteins to raise polyclonal antisera, which were subsequently purified and tested for their relative ability to inhibit erythrocyte invasion by *P. falciparum in vitro*. We used two different laboratory strains of *P. falciparum* that differed in their sensitivity to invade neuraminidase-treated erythrocytes as follows: Dd2, a strain that is dependent on sialic acid, and 3D7, which can invade through a sialic acid-independent route. The anti-*Pf*EBA175 FL and anti-*Pf*EBA175 RII sera, when tested at the same mass per volume (mg/ml) quantities, showed similar efficacy in inhibiting erythrocyte invasion by the two strains of *P. falciparum* (Fig. 6*A*). To assess whether antibodies targeting domains other than RII contributed to the inhibition of EBA175-dependent invasion, we first normalized the immunoreactivity of both antisera to RII, which revealed that anti-*Pf*EBA175 FL contained ∼5-fold less anti-RII antibodies than the anti-*Pf*EBA175 RII serum (Fig. 6*B*). After normalizing for anti-RII immunoreactivity, we observed that antibodies elicited against *Pf*EBA175 FL were able to inhibit parasite invasion more potently than those raised against the RII fragment alone, suggesting that antibodies directed against extracellular regions of *Pf*EBA175 outside of RII contribute to the ability to inhibit invasion (Fig. 6*C*).

## DISCUSSION

*P. falciparum* EBA175 has long been considered an attractive anti-malarial vaccine target because of its important role in erythrocyte invasion mediated through its interactions with GYPA expressed on the surface of host erythrocytes. The technical difficulties associated with expressing *Plasmodium* proteins recombinantly have meant that most biochemical and vaccine research has relied on expressing subfragments of the EBA175 ectodomain, most commonly the tandem DBL domains known as region II. In this study, we successfully expressed the entire full-length ectodomain of *Pf*EBA175 as a functionally active soluble recombinant protein that enabled us to perform a detailed biochemical analysis of its interaction with native GYPA, and we directly compared this with the region II subfragment.

One interesting finding from a systematic interaction screen against a panel of glycans was that the full-length *Pf*EBA175 bound Neu5Acα2–3Gal-containing glycans as expected but also interacted with Neu5Gc. These results are consistent with the observations of Orlandi *et al.* (21) who reported that the binding of native *Pf*EBA175 to erythrocytes was potently inhibited by Neu5Gc and oligosaccharides containing Neu5Acα2–3Gal. Neu5Gc is not present on human erythrocytes, due to the absence of the enzyme cytidine monophosphate-*N*-acetylneuraminic acid hydroxylase, which is required for the conversion of Neu5Ac to Neu5Gc; however, it is the predominant form of sialic acid on the erythrocytes of other apes (36). This difference in sialic acid composition has been proposed to be responsible for the restriction of *P. falciparum* and the related parasite *Plasmodium reichenowi* to their respective human and chimpanzee hosts. Martin *et al.* (35) recombinantly expressed *Pf*EBA175 RII and *P. reichenowi* EBA175 RII (*Pr*EBA175 RII) on the surface of monkey COS cells and observed binding only to human and chimpanzee erythrocytes, respectively, leading to the proposal that *Pf*EBA175 RII recognizes Neu5Ac-carrying GYPA, whereas *Pr*EBA175 RII binds Neu5Gc-containing GYPA. The findings in our study do not support this hypothesis. Further investigation of the contribution of the EBA175-GYPA interaction to the restriction of *Plasmodium* spp. parasites to their natural hosts is therefore warranted, and this will be facilitated by the ability to express the entire ectodomain of EBA175 in an active form. This is of topical interest due to the recent discovery of new *Plasmodium* species that infect gorillas and that are the closest known relatives of *P. falciparum* (40, 41).

From the six regions of homology that constitute the extracellular domain of *Pf*EBA175, only RII is known to be essential and sufficient for binding to human erythrocytes (10). Several previous studies using short chemically synthesized peptides (37, 38, 42) or *in vitro* translated EBA175 fragments (38) have suggested additional erythrocyte binding determinants outside of region II, although a recent study did not support this (12). These findings have led to the proposition of two-step binding models that invoke conformational changes in the EBA175 protein (37, 38). Our quantitative binding analysis did not find any evidence to support a conformational change in EBA175 (at least a relatively slow one that could be detected using our SPR method) but did clearly show that the full-length ectodomain of EBA175 bound native GYPA with an affinity one order of magnitude higher than the region II fragment alone suggesting that extracellular regions of *Pf*EBA175 outside of RII do play some role in the interaction with GYPA.

The region II fragment of EBA175 crystallized as an anti-parallel dimer with the putative glycan-binding sites being formed at the dimer interface, leading to the suggestion that GYPA, which forms a dimeric complex at the erythrocyte surface, induces EBA175 dimerization upon binding (18). The SPR data we obtained for the interaction of *Pf*EBA175 with GYPA fitted better to a bivalent analyte model than to a simple 1:1 binding model, which is consistent with the dimerization of EBA175 playing a role in its interaction with GYPA. Although we showed that both *Pf*EBA175 RII and *Pf*EBA175 FL were primarily monomeric in solution, both were capable of self-association, and the full-length ectodomain of EBA175 could bind itself with ∼70-fold higher affinity than the RII fragment. Extracellular regions outside of RII may therefore facilitate the interaction with GYPA by promoting homodimerization of EBA175.

Our binding analysis also revealed that soluble *Pf*EBA175 FL has a 2-fold lower affinity for Neu5Acα2–3Gal than for GYPA, but *Pf*EBA175 RII bound both with similar affinities; therefore, RII probably interacts primarily with the glycan moieties of GYPA, whereas *Pf*EBA175 FL forms contacts with both the oligosaccharides and the polypeptide backbone. We attempted to further investigate this by expressing GYPA as a recombinant protein so that it could be purified in large quantities and biochemically manipulated. Although we were able to express and purify the ectodomain of GYPA in a soluble form (rGYPA) and show that it was antigenically active by monoclonal antibody binding, it was unable to bind *Pf*EBA175 FL (data not shown). We attributed the inability of rGYPA to bind *Pf*EBA175 to under-sialylation because glycan profiling of rGYPA using a panel of lectins showed significantly lower levels of sialylation relative to native GYPA. Despite increasing the level of rGYPA sialylation by co-transfecting our GYPA expression construct with plasmids encoding an α-2,3-sialyltransferase and/or CMP-sialic acid transporter, this increase was not sufficient to confer binding to *Pf*EBA175 (data not shown). In addition, we were unable to detect any binding to native GYPA using a fragment consisting of regions III to VI of EBA175 by AVEXIS (data not shown); among other possibilities, the interaction of EBA175 with the GYPA polypeptide backbone could therefore be dependent on the binding of RII to the glycan moieties on the receptor.

In conclusion, the 10-fold higher binding affinity of *Pf*EBA175 FL for GYPA in comparison with the *Pf*EBA175 RII fragment is likely due to the participation of the extracellular regions outside of RII in the homodimerization of EBA175 and the formation of additional contacts with GYPA.

The ability to express the entire ectodomain of *P. falciparum* EBA175 as a biochemically active recombinant protein and the finding that regions of EBA175 outside of the tandem DBL domains are important for GYPA binding suggested that *Pf*EBA175 FL could be a better vaccine candidate than *Pf*EBA175 RII alone. Consistent with these observations, when normalized for immunoreactivity to RII, the antisera to *Pf*EBA175 FL was ∼5-fold more potent than the antisera to *Pf*EBA175 RII. This suggests that antibodies directed against extracellular regions of *Pf*EBA175 outside of RII also contribute to inhibiting erythrocyte invasion. We envisage that the findings reported here will contribute to a more complete understanding of the molecular basis of erythrocyte invasion by *P. falciparum* and eventually lead to the development of an effective vaccine against this infectious disease.

## Footnotes

2 The abbreviations used are:

GYPA: glycophorin-A
AVEXIS: avidity-based extracellular interaction screen
MALS: multiangle light-scattering
SEC: size exclusion chromatography
SPR: surface plasmon resonance
DBL: Duffy-binding-like
FL: full length
RII: region II
COMP: cartilage oligomeric matrix protein
HBS: HEPES-buffered saline.
